# Synergistic effects of black ginseng and aged garlic extracts for the amelioration of nonalcoholic fatty liver disease (NAFLD) in mice

**DOI:** 10.1002/fsn3.2267

**Published:** 2021-05-04

**Authors:** Guihun Jiang, Karna Ramachandraiah, Mian Anjum Murtaza, Lili Wang, Shanji Li, Kashif Ameer

**Affiliations:** ^1^ School of Public Health Jilin Medical University Jilin China; ^2^ School of Life Sciences Department of Food Science and Biotechnology Sejong University Seoul South Korea; ^3^ Institute of Food Science and Nutrition University of Sargodha Sargodha Pakistan; ^4^ Department of Food Science and Technology and BK 21 Plus Program Graduate School of Chonnam National University Gwangju South Korea

**Keywords:** aged black garlic, black ginseng, extracts, fatty liver disease, nonalcoholic

## Abstract

Nonalcoholic fatty liver disease (NAFLD) is a chronic liver disease that can lead to carcinoma, cirrhosis, and death. Since no approved medications are available, dietary interventions that include bioactive compounds have been recommended. This study investigated the effects of black ginseng extracts (BGE) and aged black garlic extracts (AGE) on high‐fat diet (HFD)‐induced obese mice. Micrograph of liver tissues of mice fed with BGE and AGE showed less lipid droplets. The BGE and AGE supplements individually and in combination lowered the marker enzymes, aminotransferase (AST), and alanine aminotransferase (ALT) levels indicating their hepatoprotective effects. Compared to the plants extracts alone, the combination of the extracts resulted in lower total cholesterol (TC) and low‐density lipoproteins cholesterol (LDL‐C), which are risk markers for cardiovascular morbidity and mortality. Diets with the combination of BGE and AGE supplements had higher superoxide dismutase (SOD), glutathione peroxidase (GSH‐Px) activities, and lower malondialdehyde indicating the synergistic effects of the extracts. Irrespective of the diet type, all treated groups showed lower tumor necrosis factor (TNF‐α) values as compared to HFD, which indicated overall immunomodulatory effect of both extracts. Therefore, the innovative formulation formed by the combination of BGE and AGE can provide hepatoprotective effects via modulating glycometabolism, lipometabolism, oxidative stress, and inflammation in mice.

## Graphical abstract

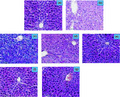

## 1 INTRODUCTION

Nonalcoholic fatty liver disease (NAFLD) is recognized as a chronic liver disease, which affects both adults and children (Puri & Sanyal, 2012). NAFLD is recognized as an end‐stage liver disease that can progressively lead to carcinoma, cirrhosis, and death (Puri & Sanyal, 2012; Younossi, 2019). The prevalence of NAFLD in the general population of the United States was estimated to be about 19.0%, whereas among adults in china, it was about 15.0% (Jia et al., 2015). Studies have shown that NAFLD is associated with obesity, diabetes mellitus (noninsulin‐dependent), and metabolic syndrome. However, not all people with NAFLD are overweight, some lean patients also have NAFLD, which is attributed to their metabolic abnormality. It is noteworthy to mention that NAFLD is a heterogeneous spectrum of diseases, which include alcoholic and nonalcoholic fatty liver diseases. Epidemiologic studies have shown that excessive consumption of alcohol causes alcoholic liver disease (ALD) (Younossi, 2019). Similarly, injury of liver has been observed seen in NAFLD patients (who predominantly have macrovesicular steatosis) as revealed by histological studies. The histological similarity between ALD and NAFLD is seen despite individuals consuming no alcohol (Puri & Sanyal, 2012).

In earlier studies, the initial cause of NAFLD was attributed to high‐fat diet and sedentary lifestyle that lead to the accretion of triglycerides (TGs) in hepatocytes. However, recently conducted investigations have shown that NAFLD is caused due to multiple issues that include lipotoxicity, oxidative stress, inflammation, and altered intestinal microbiota, which synergistically exacerbate the conditions (Lee & Lee, 2019). Despite several investigations, there is no treatment for NAFLD. As there is no FDA‐approved drug, management of this disease via lifestyle modifications that focus on diet, weight loss, and exercise has been advocated (Chalasani et al., 2017). In this regard, diets rich in nutrients and bioactive compounds have been shown to provide beneficial effects on the control of NAFLD (Dongiovanni et al., 2016). Thus, several dietary recommendations include large amounts of vegetables and fruits but exclude saturated fatty acids, trans fatty acids, and fructose. These recommendations are based on rodent models of NAFLD. In particular, plant‐derived bioactive compounds have received increased interested due to their antifibrotic, immunomodulatory, and anti‐inflammatory properties (Dongiovanni et al., 2016). While there is a wide variety of plant‐ and animal‐derived materials, certain food components possess the ability to signal and regulate major metabolic pathways that are associated with NAFLD pathogenesis (Chakravarthy et al., 2020). Consequently, increasing attention is being given to bioactive compounds that regulate the activation of genes linked to processes such as, fibrogenesis, lipid peroxidation, lipogenesis, and inflammation (Dongiovanni et al., 2016).

Black ginseng extract (BGE) is typically manufactured by steaming red or white ginseng (*Panax ginseng*) followed by drying several (nine) times. In contrast to red ginseng, black ginseng has been shown to display improved antioxidant, antihypercholesterolemic, antihyperlipidemic, antimetastatic, and anti‐inflammatory effects (Park et al., 2019). Black ginseng comprises ginsenosides, such as Rk1, Rk3, Rg3, Rg4, Rg5, Rg6, Rh1, Rh2, and Rh4. Of these ginsenosides, the most prominent are Rk1, Rg3, and Rg5 (Nam et al., 2015; Park et al., 2019). In recent years, black ginseng has been widely studied for its immunomodulation and antioxidant properties, which are due to the aforementioned bioactive compounds (Bak et al., 2014). Furthermore, it was demonstrated that fermented black ginseng treatment increased the capacity of antioxidant enzymes, such as superoxide dismutase (SOD) and catalase (CAT) in Hep‐G2 cells subjected to oxidative stress (Park et al., 2019). Recently, it was shown that BGE considerably lowered lipid accumulation and expression of lipogenic genes in diet‐induced obese mice. Owing to the ability to inhibit hepatic steatosis and control of fatty liver disease, BG is considered as a potential candidate for the treatment of liver disorders (Park et al., 2020).

Garlic, a well‐known medicinal food, possess various biological activities that include antimicrobial, antitumor, antithrombotic, and antioxidant (Maeda et al., 2019). Aged black garlic extract (AGE) is produced by extraction process using water–ethanol mixture followed by aging at room temperature. During processing, molecules that cause undesirable flavors (allicin) are converted to compounds (S‐allylcysteine (SAC), S‐allylmercaptocysteine (SAMC), and S‐1‐propenylcysteine (S1PC)) that not only have higher stability but also improved antioxidant activity (Maeda et al., 2019; Miki et al., 2017). Particularly, SAC has been shown to ameliorate NAFLD in rats with diabetes (type 2) (Takemura et al., 2013). Other benefits of AGE include improved lipid profiles and gut microbiota. In another study, fermented raw garlic at a concentration of 200 mg/kg exhibited hepatoprotective effect, which was evident in terms of reduced aminotransferase (AST) and alanine aminotransferase (ALT) levels in hepatic cells of Sprague‐Dawley rats (Tsai et al., 2019). However, the effects of black ginseng and black garlic at various doses on blood sugar, serum fat, and NAFLD have yet to be elucidated. Therefore, to the best of our knowledge, this is the first investigation of black ginseng extract (BGE) and aged black garlic extract (AGE) alone and in combination with NAFLD, serum sugar, and fats in high‐fat‐fed mice.

## 2 MATERIALS AND METHODS

### 2.1 Preparation of BGE and AGE extracts

Five‐year‐old fresh ginseng and fresh garlic were purchased from a local market in Jilin City, China. To prepare extracts, thoroughly washed fresh ginseng and garlic were placed in Korean fermentation pots (Dorim DHC‐7000C, Seoul, South Korea) for 30 hr and heated at temperatures above 80°C. Black ginseng and black garlic were then sun‐dried until final moisture content was less than 10%. Each black ginseng and garlic were weighed (40 g) and added with 1,600 ml water into fermentation pot for 18 hr to obtain BGE and ABE. The crude extract was evaporated using a rotatory evaporator and placed in a freeze dryer for 24 hr to form BGE and AGE powders. The powders were stored at ‐ 20℃ until further analysis.

### 2.2 Animals and experimental design

All animal‐related experiments performed in this study followed the procedures that were reviewed and approved by the Animal and Ethics Review Committee of Jilin Medical University of China. Seventy C57BL/6 mice were housed at a constant temperature (23°C) under artificial lighting conditions of 12:12‐hr light–dark cycle. The animals were maintained on a standard diet and water ad libitum. After 1‐week adaptation, the seventy male mice were randomly divided into 7 groups with 10 mice each. The first group was fed with a normal diet (ND, D10012G, Research Diet). The other six groups were fed a high‐fat diet (D12492: Research Diet) and denoted as follows: high‐fat diet group (HFD), HFD with 100 mg/kg.bw BGE group (HFD+BGE), HFD with 100 mg/kg.bw AGE extract group (HFD+AGE), HFD with 100 mg/kg.bw BGE and AGE mixture group (HFD+BGE+AGE100), HFD with 150 mg/kg.bw BGE and AGE mixture group (HFD+BGE+AGE150), and HFD with 200 mg/kg.bw BGE and AGE mixture group (HFD+BGE+AGE200). Each mouse was orally administrated everyday between 3:00 and 5:00 p.m. for 6 weeks. Based on standard human intake, the dosage was calculated as 100 mg/kg.bw of BGE and 100 mg/kg.bw of AGE in 1:1 ratio. Every week, mice were weighed and their growth curves were recorded.

### 2.3 Glucose and insulin tolerance test

For the glucose tolerance test, D‐glucose (2 g/kg) was injected intraperitoneally into mice that were fasted overnight for 16 hr. For the insulin tolerance test, human insulin (0.5 U/kg; Sigma) was also injected intraperitoneally into mice that were fasted for 6 hr. The blood sugar levels of mice in different dosage groups were measured at the indicated times (15min, 30min, 45min, 60min, 90min, and 120 min) using a glucometer (One Touch Ultra; LifeScan, Milpitas, CA).

### 2.4 Collection of serum and organs

At the end of the study, after fasting for 12 hr, all animals’ blood samples were collected. The collected blood samples (centrifugal tubes) were placed in a water bath thermostat at 37°C for 1 hr. The blood samples after warming were centrifuged (5,430 rpm centrifuge) at a rotational speed of 4,000 rpm for 10 min. After centrifugation, the supernatant liquid was transferred to clean centrifugal tube and kept in a refrigerator at ‐ 70°C until analysis. The collected livers were observed for liver histopathologic changes under light microscope observation with hematoxylin and eosin (H&E) staining.

### 2.5 Analytical procedure

The concentration of fasting blood glucose (FBG), total cholesterol (TC), triglycerides (TG), high‐density lipoprotein‐cholesterol (HDL‐C) and low‐density lipoprotein‐cholesterol (LDL‐C) in serum was determined with an Olympus AU2700 Clinical Chemistry Analyzer (Olympus Inc., Japan). The level of hepatic lipids, malondialdehyde (MDA), superoxide dismutase (SOD), glutathione peroxidase (GPx), T‐AOC, aspartate aminotransferase (AST), and alanine aminotransferase (ALT) was also determined through commercially available kits (Nanjing Jiancheng Bio Co., Nanjing, China).

### 2.6 Statistical analysis

Data were expressed as means ±SE. All statistical analyses were performed using SPSS software (SPSS ver. 20.0.0; SPSS Inc., Chicago, IL). Values of *p* <.05 were considered statistically significant. Differences among the groups were evaluated using two‐tailed Student's *t* tests for comparison of 2 experimental conditions or one‐way analysis of variance (ANOVA) for comparison of 3 or more experimental conditions. If ANOVA indicated significance, a Duncan post hoc test was performed.

## 3 RESULTS AND DISCUSSION

### 3.1 Glucose tolerance

**FIGURE 1:**
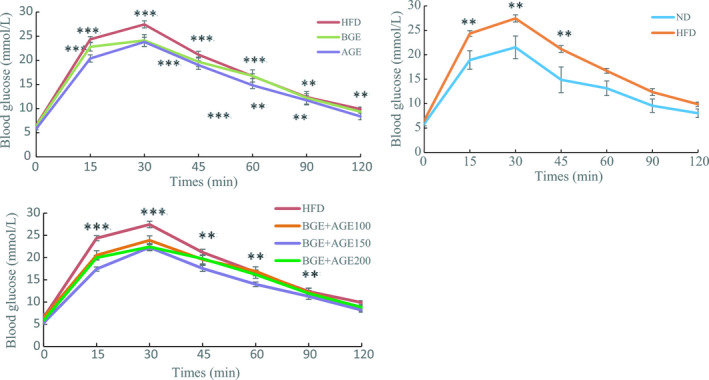
Glucose tolerance test among the different groups in C57BL/6 mice. Note: ND, normal diet group; HFD, high‐fat diet group; HF+BGE, HFD with 100 mg/kg.bw black ginseng extract group; HF+AGE, HFD with 100 mg/kg.bw aged black garlic extract group; HF+BGE+AGE100, HFD with 100 mg/kg,bw BGE and AGE mixture group; HF+BGE+AGE150, HFD with 150 mg/kg.bw BGE and AGE mixture group; and HF+BGE+AGE200, HFD with 200 mg/kg.bw BGE and AGE mixture groups. Mean ±SE (*n* = 10). Experimental values were significant difference from HFD at that time using one‐way ANOVA.*p <.05, **p <.01, ***p <.001

The results of glucose tolerance are depicted in Figure 1. At 0 min, there was no significant difference between the ND group and the HFD group (*p* >.05). However, the difference between the blood glucose value of the HFD group and that of the HFD+BGE+AGE150 was significant (*p* <.05). At 30 min, the blood glucose levels of the ND, HFD, and treatment groups reached the maximum, whereas the values for the HFD+BGE+AGE150 and HFD+BGE+AGE200 groups were significantly lower. After 30 min, the blood glucose of all groups was decreased. At 120 min, the blood glucose value of each group tended to be stable. At this time, there was a significant difference between the blood glucose value of the HFD group and the HFD+AGE group, the HFD+BGE+AGE150 group. These results suggested that both BGE and AGE led to improved oral glucose tolerance in the HFD‐fed mouse group and ameliorated the glycolipid homeostasis. In a study by Chae et al., (2019), area under the blood glucose from 0 to 180 min (AUG) was reported to be lowered in mice fed with ginseng berry extract. Owing these effects, increased attention is being given to ginsenosides for long‐term glycemic control. In another study when AGE was administered, the serum glucose levels were found to be lowered in hyperglycemic mice (Maeda et al., 2019). In addition, Seo et al., (2012) suggested that intake of AGE lowered homocysteine levels in postmenopausal women. Hence, it may be implied that AGE may also exert effect on blood glucose value. Thus, it is possible that bioactive components of ginseng and garlic play major roles in regulation of peroxisome proliferator‐activated receptor coactivator‐1α (PGC1 α), CCAAT/enhancer‐binding protein (CEBP), and AMP‐activated protein kinase (AMPK), which consequently regulate the hepatic steatosis in the HFD mouse groups (Chae et al., 2019).

### 3.2 Insulin tolerance

**FIGURE 2:**
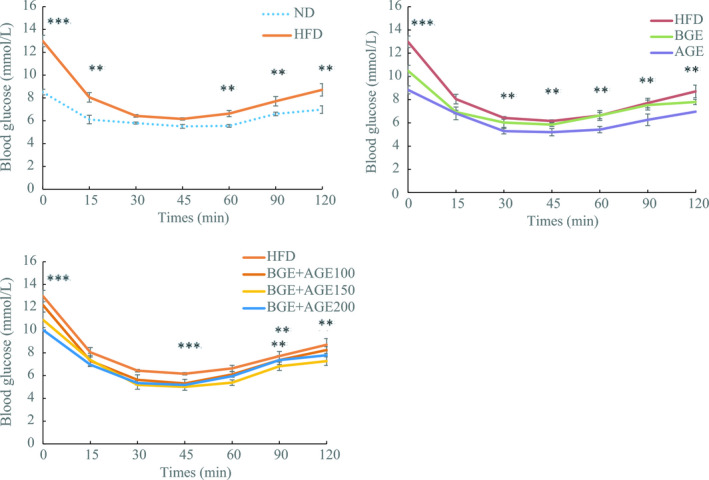
Insulin tolerance test in different groups in C57BL/6 mice. Note: ND, normal diet group; HFD, high‐fat diet group; HF+BGE, HFD with 100 mg/kg.bw black ginseng extract group; HF+AGE, HFD with 100 mg/kg.bw aged black garlic extract group; HF+BGE+AGE100, HFD with 100 mg/kg,bw BGE and AGE mixture group; HF+BGE+AGE150, HFD with 150 mg/kg.bw BGE and AGE mixture group; and HF+BGE+AGE200, HFD with 200 mg/kg.bw BGE and AGE mixture groups. Mean ±SE (*n* = 10). Experimental values were significant difference from HFD at that time using one‐way ANOVA.*p <.05, **p <.01, ***p <.001

The comparison of mean blood glucose level between each group showed significant differences (*p* <.05) (Figure 2). At the beginning of 0 min, the blood glucose value of each group showed a downward trend. After 45 min, the blood glucose value of each group reached the lowest value and then showed an upward trend. At this time, the blood glucose value of the HFD group was statistically significant (*p* <.05) compared with that of the AGE group, the HFD+BGE+AGE100 group, the HFD+BGE+AGE150 group, and the HF+BGE+AGE200 group. At 120 min, the blood glucose value of each group was stable. At this time, the blood glucose value of the HFD group was significantly different from that of the AGE group and the HFD+BGE+AGE150 group (*p* <.05). These differences in the HFD‐fed mouse group, the AGE‐fed group, and the HFD+BGE+AGE150 groups might be due to the massive secretion of insulin in the HFD‐fed group. Hence, it was evident that AGE and HFD+BGE+AGE150 caused the reduction in serum glucose by rendering the promotion of glucose update and improvement in insulin sensitivity. Both BGE and AGE possess propionic acid, which has been reported to play regulatory roles in lowering serum lipid and glucose levels. Furthermore, numerous published articles have endorsed the roles of propionic acid in the regulation of gut microbiota and relieving inflammation (Wu et al., 2020; Zhang et al., 2019). Hence, it is suggested that BGE and AGE can improve glycolipid metabolism by affecting gut microbiota. Zhang et al., (2019) have reported similar findings. Another study by Wang et al., (2016) also reported that phenolic compounds exert positive effects on gut microbes and lead to improvement in hepatic fat deposition, oxidative stress, insulin resistance, and intestinal inflammation in mice. Likewise, Li et al., (2019) observed inhibition of NAFLD in high‐fructose‐fed mice due to phenolic compounds derived from loquat fruit indicating its antihyperlipidemic and antihyperglycemic potentials.

### 3.3 ALT, AST, and liver lipids (TG, TC, HDL‐C, and LDL‐C)

The effects of different dietary supplementations on ALT and AST are shown in Table 1. The highest serum ALT value (48.75 U/g prot) in mice was observed in the HFD group as compared to ND (36.00 U/g prot) or other groups. However, the lowest serum ALT value was observed in the HFD+AGE (33.75 U/g prot) group indicating the hepatoprotective effect of AGE supplementation. Similarly, hepatoprotective effects were also observed in groups treated with HFD+BGE (34 U/g prot), HFD+BGE+AGE150 (37.00 U/g prot), HFD+BGE+AGE100 (38.00 U/g prot), and HFD+BGE+AGE200 (39.00 U/g prot). As compared to the serum AST value of control (127.88 U/g prot), the highest serum AST value was observed with the HFD group (142.38 U/g prot) in mice. Contrarily, the lowest serum AST value was observed in the group treated with HFD+AGE (125.00 U/g prot). This also showed that the hepatoprotective effect was most prominent for the HFD+AGE group followed by HFD+BGE+AGE150 (127.88 U/g prot), HFD+BGE (130.00 U/g prot), HFD+BGE+AGE200 (134.51 U/g prot), and HFD+BGE+AGE100 (138.00 U/g prot). It was evident that, while high‐fat diet induced the increases in marker enzymes for liver function (ALT and AST), nutritional interventions (HFD+BGE and HFD+AGE) led to marked decreases in these liver enzymes, thereby ameliorating the damaging effects of NAFLD. It is likely that the improvement of liver function could be due to the hepatoprotective effects of Rb1 and CK ginsenosides along with Rg3 and Rh2, which act against liver injuries caused by tert‐Butyl hydroperoxide (Kim, 2016).

**TABLE 1:** ALT and AST activities of mice treated with BGE and AGE

Groups	ALT (U/g prot)	AST (U/g prot)
ND	36.00 ± 4.60bc	127.88 ± 11.48ab
HFD	48.75 ± 3.54a	142.38 ± 14.92a
HFD+BGE	34.00 ± 3.59c	130.00 ± 13.46b
HFD+AGE	33.75 ± 2.49c	125.00 ± 14.27ab
HFD+BGE+AGE100	38.00 ± 3.16bc	138.50 ± 14.47ab
HFD+BGE+AGE150	37.00 ± 4.21bc	127.88 ± 10.87ab
HFD+BGE+AGE200	39.00 ± 6.07b	134.50 ± 15.40ab

Note

Experimental values were compared with those for the high‐fat diet group using Duncan's multiple comparison test after one‐way ANOVA. *p* <.05.

^a‐d^denoting a significant post hoc difference from the other groups (*p* <.05).

TG, TC, HDL‐C, and LDL‐C of mice treated with BGE and AGE are shown in Table 2. The serum level of TG in HFD mice was higher than that of other groups (*p* <.05). BGE (Park et al., 2020) and AGE (Maeda et al., 2019) have been reported to exhibit profound effect on the regulation of lipid metabolism. This could be attributed to the increased carnitine content in BGE and AGE. Carnitine is known to be involved in the β‐oxidation of fatty acids in mitochondria. It is likely that the higher carnitine levels in BGE and AGE could have enhanced the body lipolysis (Zhang et al., 2019). In published literature, it is suggested that ginseng plays a major role in amelioration of taurine and hypotaurine metabolism, in turn leading to lipid‐lowering effects. Both taurine and hypotaurine have their pertinent roles in regulating glucose‐lipid homeostasis and maintenance of cardiovascular function (Murakami, 2014). However, exact mechanistic details have yet to be investigated.

**TABLE 2:** Blood fat of mice treated with ginseng and garlic extracts

Groups	TG (mmol/gprot)	TC (mmol/gprot)	HDL‐C (mmol/gprot)	LDL‐C (mmol/gprot)
ND	1.78 ± 0.19b	3.16 ± 0.17d	4.35 ± 0.23a	1.39 ± 0.21c
HFD	2.96 ± 0.23a	5.32 ± 0.29a	2.53 ± 0.21d	2.39 ± 0.44 a
HFD+BGE	1.95 ± 0.36b	4.89 ± 0.33bc	3.78 ± 0.30bc	1.93 ± 0.23b
HFD+AGE	1.74 ± 0.36b	4.65 ± 0.29c	3.56 ± 0.30c	0.99 ± 0.28b
HFD+BGE+AGE100	1.74 ± 0.25b	5.14 ± 0.33ab	3.87 ± 0.20b	1.92 ± 0.22b
HFD+BGE+AGE150	1.44 ± 0.23c	4.87 ± 0.41bc	4.22 ± 0.34a	1.61 ± 0.43bc
HFD+BGE+AGE200	1.66 ± 0.20bc	4.91 ± 0.27bc	3.77 ± 0.35bc	1.76 ± 0.44b

Note

Experimental values were compared with those for the high‐fat diet group using Duncan's multiple comparison test after one‐way ANOVA. *p* <.05.

^a‐d^denoting a significant post hoc difference from the other groups (*p* <.05).

As shown in Table 2, the HFD group had the highest TC, whereas all other groups showed lower TC (*p* <.05). In a study, it was suggested that diet‐based modality in rats may lead to regulation of glycemic response and amelioration of insulin sensitivity, which could exert potential impact on weight loss and lowering of serum lipid levels (Chae et al., 2019). Apart from insulin resistance, the incidence of T2DM is also closely related to increased levels of serum TC, TG, and LDL‐C. Moreover, the threat caused by dyslipidemia is of more significance than T2DM threat alone (Murakami, 2014). However, the results of current study suggested that BGE and AGE could play an important role in the amelioration of hyperlipidemia caused by metabolic disorders in diabetics. These results were consistent with the findings of Wu et al., (2020).

Similar to LDL‐C, the HDL‐C levels in the ND group were significantly different from that of the HFD group (*p* <.05). Each group, BGE, AGE, and HFD+BGE+AGE200 group, was significantly lower than the HFD group, respectively (*p* <.05). It has already been established that glucose and lipid metabolisms are closely interlinked with each other. It is noteworthy that low HDL‐C and hypertriglyceridemia can both result and cause disturbed glucose metabolism (Parhofer, 2015). However, the current study showed the enhanced potential of BGE and AGE in improving serum lipid regulation of HFD mice. In particular, the combination of BGE and AGE (HFD+BGE+AGE150) exhibited superior TG, LDL lowering, and HDL increasing effects as compared to the plant extracts individually. This indicated the synergistic effect of the two extracts, which are rich sources of nutrients and polyphenols (Dongiovanni et al., 2016). Similar lipid‐lowering effects caused by loquat fruit extract were reported in a study (Li et al., 2019). Moreover, the plant‐based polyphenolic compounds have also been associated with the prevention of hyperlipidemia. Both BGE and AGE have rich phenolic‐rich nutritional profiles, which exert beneficial effect on serum TC, TG, HDL‐C, and LDL‐C. In particular, TC and LDL are the biomarkers of the harmful accumulation of cholesterol in body (Saba et al., 2016). Nonetheless, the lipid‐lowering effects of BGE and AGE could be attributed to the downregulation of key genetic markers responsible for fat metabolism, such as 3‐hydroxy‐3‐methyl‐glutaryl‐CoA reductase (HMG‐CoAr), acetyl‐coenzyme A (CoA) acetyltransferase 2 (ACAT2), and sterol regulatory element‐binding protein 2 (SERBP2) (Lee et al., 2013) In one study, it was reported that BGE lowered the LDL and TC levels in mice, whereas HDL remained unaffected (Saba et al., 2016). In contrast, this study for the first time showed the alleviation effect of both BGE and AGE on hypercholesterolemia by lowering TC and LDL‐C.

### 3.4 Antioxidant activity

While SOD and GSH‐Px of the HFD group were significantly lower, MDA content was significantly higher than that of the ND group (Table 3). Furthermore, GSH‐Px of HFD+BGE+AGE100 and HFD+BGE+AGE150 was significantly higher than the HFD group (*p* <.05). SOD of HFD+BGE+AGE150 and HFD+BGE+AGE200 was significantly higher than the HFD group, but for the same diet groups, MDA was lower (*p* <.05). The results showed that the combination of BGE and AGE increased the activity of SOD and GSH‐Px in liver tissues and in turn decreased the content of MDA. Although the combination of BGE and AGE displayed antioxidant capacity, at optimal concentrations, the antioxidant capacity was improved. Particularly, the HFD+BGE+AGE200 group showed the highest SOD and the lowest MDA, thereby indicating a synergistic effect at this concentration. In a human study, it is shown that *Citrus bergamia* and *Cynara cardunculus* extracts in combination produced synergistic effects, which caused lowered MDA while SOD and GPx were increased (Musolino et al., 2020). However, it is important to note that oxidative stress caused by the production of reactive oxygen species (ROS) is neutralized by enzymatic and nonenzymatic antioxidants. However, in the case of diabetes, processes such as mitochondrial respiratory chain reaction, glucose autoxidation, and glycosylation can cause an increase in the intracellular level of ROS (Patar et al., 2018). Additionally, the high ROS generation is also linked to the activation of stress‐sensitive signaling pathways that are triggered by mitogen‐activated protein kinases (MAPK) (Li et al., 2019). Moreover, the MDA is an indicative of the cellular damage caused by free radicals. Hence, SOD, GHS‐Px, and MDA activities in the ND, BGE, and AGE groups were quantified for the evaluation of oxidative stress. Nonetheless, the results of this study imply that BGE and AGE can induce the promotion of ROS scavenging. These results are in agreement with the findings of a previous research by Wu et al., (2020).

**TABLE 3:** SOD, GSH‐Px, MDA, and T‐AOC content of liver tissues from mice treated with ginseng and garlic extracts

Groups	SOD (U/mgprot)	GSH‐Px (U)	MDA (nmol/mgprot)	T‐AOC (U/g)
ND	1,035.960 ± 89.725ab	387.304 ± 29.795bc	1.640 ± 0.299e	0.080 ± 0.007a
HFD	952.589 ± 26.953b	335.810 ± 32.539d	3.783 ± 0.430a	0.057 ± 0.008b
HFD+BGE	950.372 ± 104.813b	387.295 ± 50.471bc	2.099 ± 0.250 cd	0.058 ± 0.006b
HFD+AGE	997.544 ± 75.774b	353.229 ± 34.725 cd	3.158 ± 0.152b	0.076 ± 0.004a
HFD+BGE+AGE100	978.384 ± 76.426b	404.292 ± 31.156b	2.418 ± 0.169c	0.077 ± 0.011a
HFD+BGE+AGE150	980.389 ± 68.907b	532.477 ± 60.102a	2.368 ± 0.639c	0.080 ± 0.012a
HFD+BGE+AGE200	1,109.075 ± 69.981a	383.500 ± 56.259bc	1.712 ± 0.573de	0.066 ± 0.007b

Note

Experimental values were compared with those for the high‐fat diet group using Duncan's multiple comparison test after one‐way ANOVA.*p* <.05.

^a‐e^denoting a significant post hoc difference from the other groups (*p* <.05).

The results of T‐AOC are also presented in Table 3. It was evident that high‐fat diet (HFD) was associated with lower liver antioxidant capacity, while inclusion of plant extracts in diets (HFD+BGE+AGE150 and HFD+BGE+AGE100) led to significantly higher liver antioxidant (T‐AOC) capacity, thereby ameliorating the damaging effects of NAFLD. Similar findings have been reported in a study, wherein emodin, a plant‐derived bioactive compound, influenced T‐AOC (Cui et al., 2014). The variations in T‐AOC levels suggested that liver pro‐antioxidant balance is necessary to carry out the removal of ROS and changes in liver antioxidant enzymes are often perceived as biomarker of antioxidant response. However, in this study, synergistic effect was observed when the plant extracts were combined (HFD+BGE+AGE150). Along with GSH and SOD, T‐AOC also demonstrates the protective roles of plant extracts in the amelioration of oxidative damage effects. These results suggested that both BGE and AGE might provide antioxidant activity, promote hepatic glycogen synthesis, and reduce serum glucose levels (Wang et al., 2019). As the incidence of NFLD is closely linked to increased inflammation, BGE and AGE might also lower the oxidative stress‐mediated systemic inflammation in body, thereby preventing the onset of NAFLD (Li et al., 2019). However, the combination of BGE and AGE can result in an innovative formulation, which could be utilized in the treatment of NAFLD.

### 3.5 IL‐6 and TNF‐α

Interleukin 6 (IL‐6) and tumor necrosis factor (TNF‐α) have been reported as pro‐inflammatory cytokines, which play major roles in the development of systemic inflammation. The effects of BGE and AGE supplementation on IL‐6 and TNF‐α are presented in Table 4. The ND group showed IL‐6 expression value of 96.088 (pg/mL), whereas the HFD group showed the highest IL‐6 value (109.93 pg/mL) in mice. The high IL‐6 value observed in the high‐fat‐fed group indicated the fat‐induced inflammation in mice. On the other hand, HFD+BGE+AGE200 (92.686 pg/mL) was the most effective in lowering the IL‐6 values followed by groups treated with HFD+BGE+AGE150 (96.576 pg/mL), HFD+BGE (98.895 U/g), and HFD+BGE+AGE100 (102.519 U/g). As compared to the plant extracts alone, the combination of plant extract supplements at the highest concentration (HFD+BGE+AGE200) resulted in the lowest IL‐6 value.

**TABLE 4:** IL‐6 and TNF‐α content of liver tissues from mice treated with ginseng and garlic extracts

Groups	IL−6 (pg/mL)	TNF‐α (pg/mL)
ND	96.088 ± 7.601bc	107.932 ± 10.566d
HFD	109.930 ± 10.409a	141.521 ± 3.152a
HFD+BGE	98.895 ± 8.209bc	130.558 ± 4.646b
HFD+AGE	102.008 ± 9.115ab	96.062 ± 7.238e
HFD+BGE+AGE100	102.519 ± 8.009ab	107.579 ± 8.322d
HFD+BGE+AGE150	96.576 ± 6.451bc	119.930 ± 9.064c
HFD+BGE+AGE200	92.686 ± 6.308c	130.001 ± 6.059b

Note

Experimental values were compared with those for the high‐fat diet group using Duncan's multiple comparison test after one‐way ANOVA. *p* <.05.

^a‐e^denoting a significant post hoc difference from the other groups (*p* <.05).

Similar to IL‐6, Table 3 presents the values of TNF‐α, which was 107.932 (pg/mL) for the control group. The highest TNF‐α value (141.521 pg/mL) was seen with the HFD group (*p* <.05). These results clearly indicated that high‐fat diet could lead to higher induced inflammation in mice. However, the lowest TNF‐α value was observed in the group treated with HFD+AGE (96.062 pg/mL). Other diet‐fed groups, such as HFD+BGE+AGE100, HFD+BGE+AGE150, and HFD+BGE+AGE200, showed TNF‐α expression values of 107.579, 119.93, and 130.01 pg/mL, respectively. Both HFD+BGE and HFD+BGE+AGE200 showed similar TNF‐α value of 130.5 pg/mL. Regardless of the diet type, all treated groups showed significantly lower TNF‐α values as compared to the HFD group, which was indicative of the overall immunomodulatory effect of black ginseng and aged garlic in treated mice. These results are in agreement with a report by Lu et al., (2009), wherein ginsenosides that include Rg3, Rh2, Re, Rb1, and Rg1 exhibit significantly higher anti‐inflammatory and antioxidant properties. In particular, Rb1 has been linked with the reduced release of IL‐6 and TNF‐α in carcinogenic rats suffering from bone pain. In addition, the ginsenosides have been reported to inhibit the expression of pro‐inflammatory cytokines (IL‐6 and TNF‐α) in liver and adipose tissues of HFD mice. Furthermore, studies have shown that ginseng extract is associated with the enhancement of killer cell activities, interferon production, phagocytosis, and partial regulation of host defense mechanisms (Chen et al., 2015).

### 3.6 Histopathological studies of the liver

**FIGURE 3:**
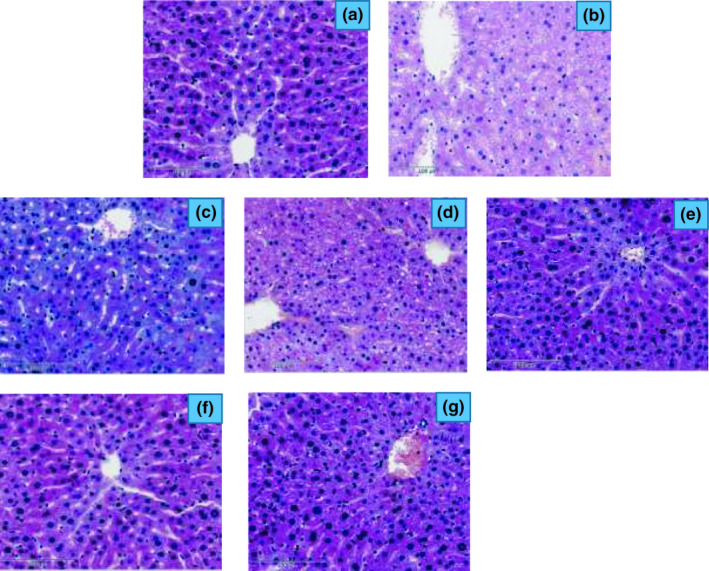
Histopathological of liver cell of mice treated with ginseng and garlic extracts. Note: (a) ND, normal diet group; (b) HFD, high‐fat diet group; (C) HF+BGE, HFD with 100 mg/kg.bw black ginseng extract group; (d) HF+AGE, HFD with 100 mg/kg.bw aged black garlic extract group; (E) HF+BGE+AGE100, HFD with 100 mg/kg,bw BGE and AGE mixture group;(f) HF+BGE+AGE150, HFD with 150 mg/kg.bw BGE and AGE mixture group; and (G) HF+BGE+AGE200, HFD with 200 mg/kg.bw BGE and AGE mixture groups

The morphological characteristics of liver tissues of all experimental groups are shown in Figure 3. The photomicrograph demonstrated the histopathological observation in the ND, HFD, and BGE/AGE groups. The photomicrographs demonstrated that no lipid droplets were found in liver tissues of mice in the ND group. However, in the HFD group, lipid droplets were found in mice liver tissues, and some cells or even nuclei were squeezed to one side. Although lipid droplets were observed in the extract (BGE/AGE) intervention groups, the degree of steatosis was not as high as the HFD group. The results showed significant pathological modification in obese and diabetic rats administrated with the BGE and AGE groups. Hence, it could be implied that BGE and AGE may cause the regulation of glucose homeostasis through different mechanisms that exert positive effects on the improvement of pancreas islet function and glucose tolerance in diabetic mice.

## 4 CONCLUSION

The present study showed that both BGE and AGE led to improved oral glucose tolerance in the HFD‐fed mouse group and ameliorated the glycolipid homeostasis. Micrographs demonstrated the presence of lipid droplets, which were squeezed to one side in the liver tissues of HFD mice. While lipid droplets were present in the BGE/AGE group, the degree of steatosis was not as high as that observed in the HFD group. Based on the blood glucose test, the glucose value of the AGE group increased the slowest, whereas the HFD group increased the fastest. High‐fat diet induced the increases in marker enzymes of liver function (ALT and AST), whereas the plant extract‐supplemented diets (HFD+BGE and HFD+AGE) led to marked decreases in these liver enzymes, thereby ameliorating the damaging effects of NAFLD. The combination of plant extracts (BGE+AGE) synergistically showed substantial lipid‐lowering effects as displayed by the lower TG, TC, and LDL‐C. Increased SOD, GSH‐Px, and T‐AOC and decreased MDA observed with the combination of the extracts than the extracts alone indicate their synergistic effects. It is likely that the compounds from the two extracts caused cumulative effects. Irrespective of the diet type, all treated groups showed significantly lower TNF‐α values than the HFD group, indicating the overall immunomodulatory effect of black ginseng and aged garlic in treated mice. Therefore, both BGE and AGE with high anti‐inflammatory, antioxidant properties along with lipid‐lowering, and hepatoprotective effects can be utilized in the amelioration of NAFLD.

## CONFLICT OF INTEREST

The authors declare that they have no known competing interests.

## Data Availability

The data used to support the findings of this study are available from the corresponding author upon request.
